# *In situ* measurement of autophagy under nutrient starvation based on interfacial pH sensing

**DOI:** 10.1038/s41598-018-26719-4

**Published:** 2018-05-29

**Authors:** Toshiya Sakata, Akiko Saito, Haruyo Sugimoto

**Affiliations:** Department of Materials Engineering, School of Engineering, The University of Tokyo, 7-3-1 Hongo, Bunkyo-ku, Tokyo, 113-8656 Japan

## Abstract

In this study, we report a novel method for the *in situ* measurement of autophagy under nutrient starvation using a principle of semiconductor technology. A semiconductor-based field-effect transistor (FET) biosensor enables the direct detection of ionic or molecular charges under biological conditions. In particular, cellular respiration accompanied by the generation of carbon dioxide can be continuously and directly monitored as a change in pH at a cell/sensor interface. When autophagy was induced in HeLa cells on a FET biosensor under nutrient starvation, the surface potential increased more significantly for about 15 h than that for nonstarved cells. This positive shift indicates an increase in the number of hydrogen ions produced from the respiration of starved cells because the sensing surface was previously designed to be sensitive to pH variation. Therefore, we have found that cellular respiration is more activated by autophagy under nutrient starvation because the amino acids that decomposed from proteins in autophagic cells would have been rapidly spent in cellular respiration.

## Introduction

For living cells, nutrient depletion may stimulate various functions. Simply, apoptosis may be induced in cells by nutrient starvation. However, regulated and adapted functions are provided for cells such as autophagy under nutrient starvation. Autophagy is an intracellular degradation system, by which cytoplasmic contents are degraded in lysosomes, and it is also dynamically induced by nutrient depletion to provide necessary amino acids within cells such as yeasts, thus helping them adapt to starvation, which has been reported to be essential for cellular homeostasis^1,2^. Therefore, metabolism such as cellular respiration in a cell may be activated to temporarily prevent apoptosis through autophagy. Recently, autophagy has been studied vigorously for various physiological processes, such as cellular remodeling^3,4^, differentiation^5^, the production of a pulmonary surfactant^6^, and nonapoptotic cell death during embryogenesis^7^. Defective autophagy may contribute to the pathogenesis of mammary tumors^8^ and a specific type of myopathy^9,10^, and autophagy-defective (*atg*) mutants of yeast are very sensitive to extracellular pH and defective respiration leads to cell death in the *atg* mutants under starvation^11^. Cellular respiration involves a series of metabolic reactions to produce adenosine triphosphate (ATP) by the uptake of nutrients such as glucose and oxygen, followed by the release of waste products such as carbon dioxide. In particular, the mitochondria play an important role in cellular respiration to produce ATP via the citric acid cycle, electron transfer system, and oxidative phosphorylation in aerobic respiration, the degradation of which has recently been focused on in relation to various conditions such as diabetes, Alzheimer’s disease, and aging^12–14^. In aerobic respiration, carbon dioxide dissolves in a solution, resulting in the generation of hydrogen ions, which is observed as a change in pH. Thus, *in situ* respiratory monitoring of autophagy based on pH changes should contribute to the elucidation of various diseases and pharmaceutical discoveries.

As a method of cell analysis, fluorescence imaging is generally used by labeling cells with fluorescent dyes. We can clearly observe a specific targeted molecules on/in cells by fluorescence microscopy. However, this method has some disadvantages such as a lack of quantitative information and the photobleaching of fluorescent dyes, which affects the long-term monitoring of cells. In particular, some fluoresceins are often used as pH indicators, but they do not easily enable long-term monitoring of changes in pH on the cell surface. Recently, semiconductor-based field effect transistor (FET) biosensors have been studied and developed for application to clinical diagnosis, drug discovery, tissue engineering, and so forth^15–23^. A FET biosensor can continuously detect molecular recognition events that are accompanied by changes in charge density without the need for labeled materials for a long time^17–19^ and be easily arrayed using conventional semiconductor processes to measure multiple samples^20^. Moreover, the electrical signals obtained from FET devices enable direct and quantitative analyses of biosamples. Electrical charges of ions or biomolecules interact electrostatically with electrons in the channel of semiconductor device. Therefore, ionic behaviors based on biological phenomena can be directly and quantitatively detected in real time using semiconductor devices. Most *in vivo* biological phenomena are closely related to charged media, for example, DNA molecules with negative charges based on phosphate groups, ions such as Na^+^ and K^+^ passing through ion channels in the cell membrane to maintain homeostasis, and so forth.

In our previous works, the respiration activities of pancreatic β cells of a rat, a single mouse embryo, and bovine chondrocytes on a gate were monitored noninvasively, quantitatively, and continuously as the change in pH using the principle of FET biosensors^21–23^. Since the gate insulator usually consists of an oxide with hydroxyl groups at the surface in a solution, FET biosensors are sensitive to concentration changes of hydrogen ions with positive charges and consequently can be utilized as pH sensors. Thus, the pH variation due to cellular respiration activities can be monitored at a cell/gate interface on the basis of carbon dioxide generated by cellular respiration and dissolved in a medium. That is, a change in pH at the nanogap between the cells and the oxidized gate of FET biosensors is the basis for the quantitative and real-time measurement of living cells. As one of the cellular events for *in situ* monitoring of autophagy, we focused on cellular respiration, which can be detected as a change in pH, because autophagy under nutrient starvation is closely related to cellular metabolic processes such as respiration.

In this paper, we report a novel method for the *in situ* measurement of autophagy under nutrient starvation using a FET biosensor. In particular, we focus on the cell/gate interfacial pH resulting from the cellular respiration activity of starved cells because the FET biosensor can directly detect pH variation on the basis of the field effect.

## Results

### Conceptual structure of semiconductor-based FET biosensor

The principle of the semiconductor-based FET biosensor is based on the potentiometric detection of changes in charge density on a gate insulator (Fig. S1(a)), which is composed of insulated layers of Ta_2_O_5_/Si_3_N_4_/SiO_2_ on a silicon substrate. The surface of an oxide such as Ta_2_O_5_ is covered by hydroxyl groups in attaching with a buffer solution, which are very sensitive to hydrogen ions (Fig. S1(b)). The responsivity of FET biosensors to hydrogen ions is well known to exhibit a Nernstian response (about 59 mV/pH at room temperature); such FETs acting as pH sensors are called ion-sensitive field-effect transistors (ISFETs)^15^. According to an electrical signal obtained with our ISFETs, the surface potential at a Ta_2_O_5_ gate surface actually shifted by about 58 mV/pH (Fig. S1(c)). This result is typical for ISFETs, which were utilized as FET biosensors with cultured cells to monitor cellular respiration activity in this study^21–23^.

**Figure 1:**
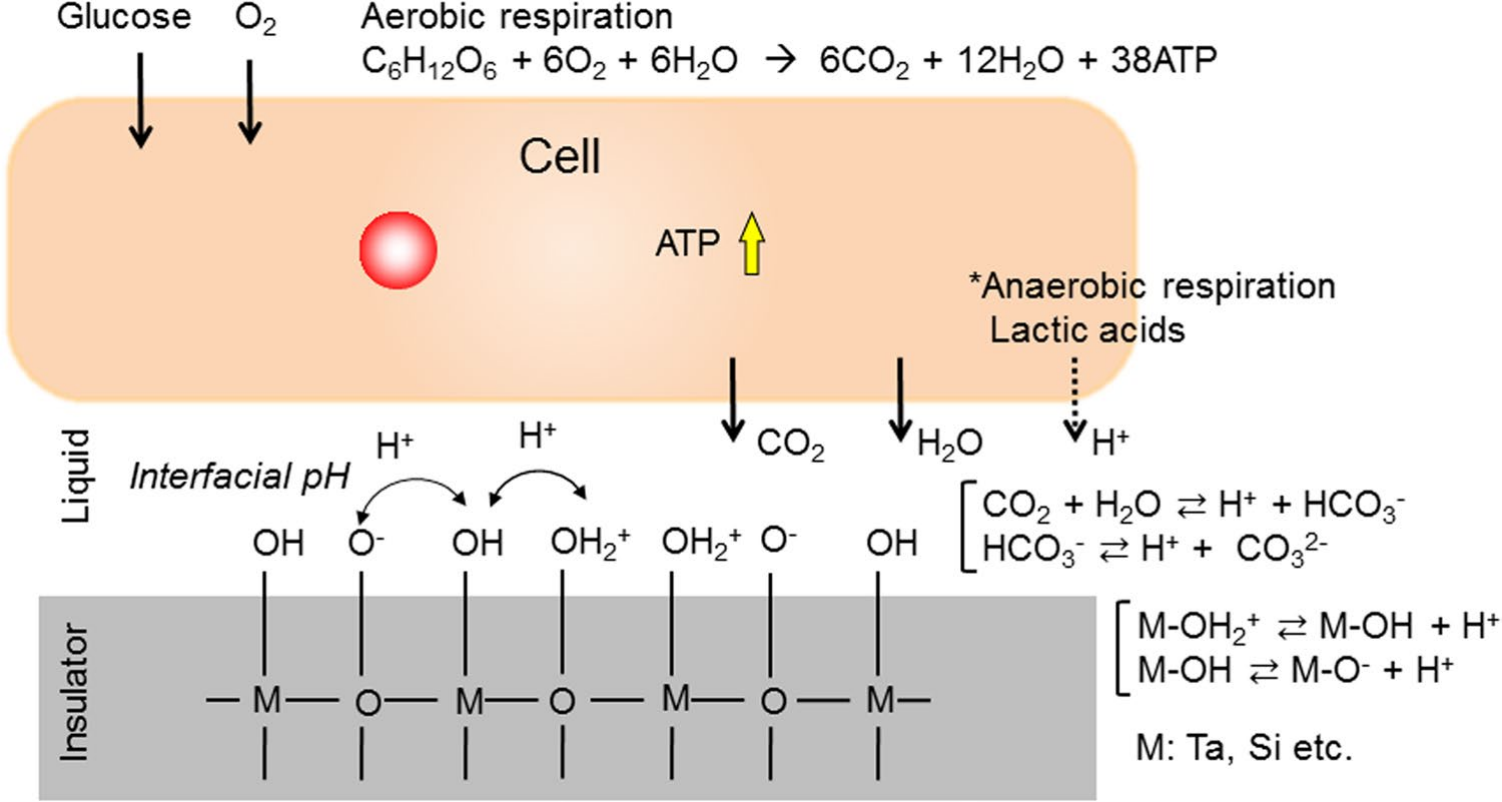
Concept of interfacial pH detection based on cellular respiration activity. An oxide gate insulator acting as the gate of a FET biosensor has hydroxyl groups at the surface in an aqueous solution. The hydroxyl groups undergo an equilibrium reaction with hydrogen ions depending on the pH. In general, cellular respiration generates adenosine triphosphate (ATP) in the case of aerobic respiration, which transports chemical energy within the cells for metabolism, then carbon dioxide (CO_2_) is released from the cells. CO_2_ is dissolved into the solution around the cell/gate interface, resulting in a change in interfacial pH. On the other hand, lactic acid may contribute to the change in interfacial pH in the case of anaerobic respiration.

HeLa cells were satisfactorily cultured on a gate surface in the same way as on a conventional culture dish. Here, we need to focus on the charge behaviors of ions and biomolecules at the interface between the cell membrane and gate surface, which are different from those in a bulk solution, to directly detect cell functions. This is because cells are alive and their functions should be directly monitored in real time. That is, the “interfacial pH” at the cell/gate interface reflects cellular functions *in situ* (Fig. 1). In fact, previous works showed a gap of 10 nm order at the cell/substrate interface by total-internal-reflection fluorescence microscopy^24,25^. The interfacial pH is considered as the localized pH at the cell/gate interface and depends on the living state of the cells^22^. Therefore, we can expect that the cellular respiration activity can be monitored as the change in the interfacial pH because in aerobic respiration carbon dioxide dissolves continuously into the solution, resulting in the generation of hydrogen ions at the cell/gate interface or lactic acids are released there in anaerobic respiration.

### Electrical measurement of autophagy

**Figure 2:**
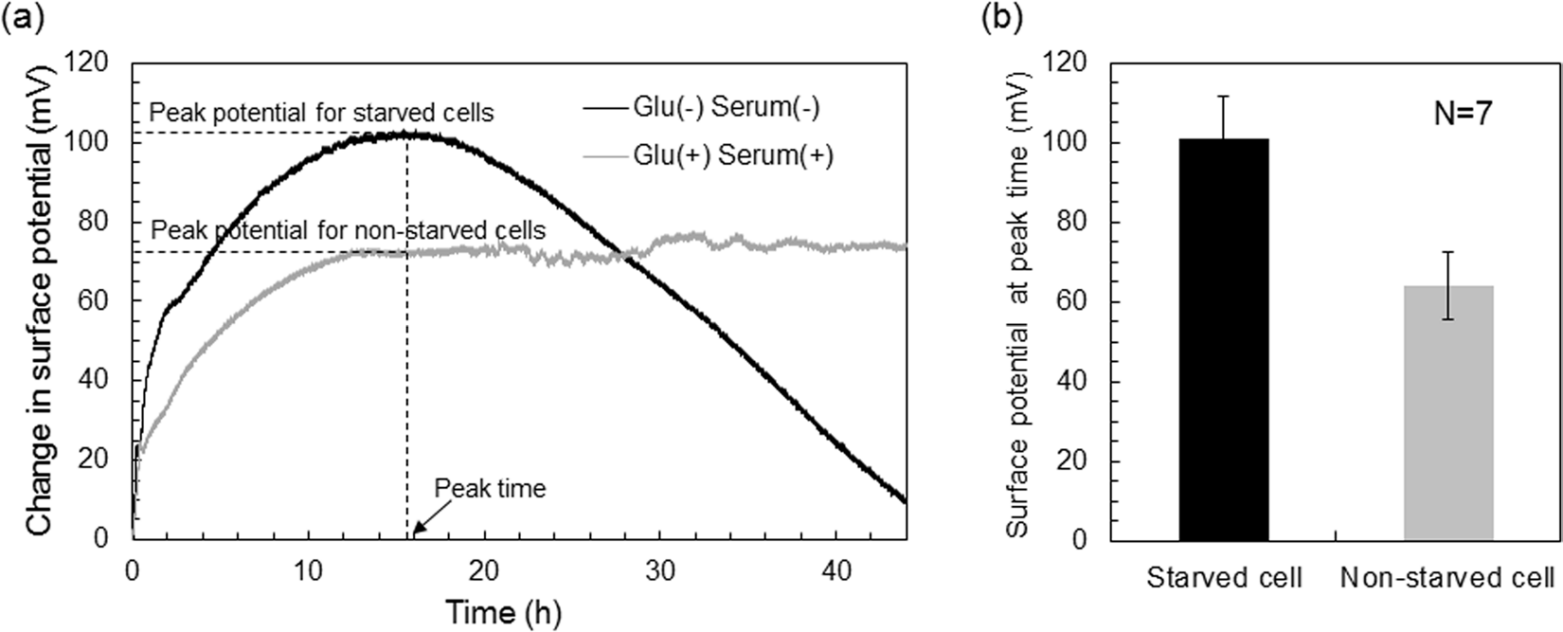
(**a**) Change in surface potential of FET biosensor for starved and non-starved cells. The gray line shows the electrical signal of non-starved cells in a culture medium including glucose and serum. The black line shows the electrical signal of starved cells in a culture medium without glucose and serum. The culture medium was changed with the appropriate one at 0 h. The peak time was defined as the time showing the peak potential for the starved cells, then the peak potential for the non-starved cells was also determined at the peak time. (**b**) Difference between surface potentials for starved and non-starved cells at peak time. These data were obtained by performing seven measurements for both starved and non-starved cells. There were significant differences between the two groups (p < 0.05).

The metabolism of starved cells can be detected in real time using a FET biosensor because the interfacial pH between the cell and the gate of the FET biosensor is measured as the gate surface potential on the basis of the field effect (Fig. 2). Figure 2(a) shows the change in the surface potential of a FET biosensor for the incubation time after changing the culture medium with a fresh one. The surface potential for HeLa cells after exchanging the original culture medium for one without serum and glucose increased more than that after exchanging the original culture medium for a prepared medium containing both substances. This positive shift indicates an increase in the number of positive charges based on hydrogen ions, which were generated by the dissolution of carbon dioxide, because the ISFET was very sensitive to pH variation. Therefore, this means that greater cellular respiration activity was induced under nutrient starvation, which should trigger autophagy in a cell. That is, the amino acids generated by the decomposition of proteins in autolysosome are utilized for cellular respiration under nutrient starvation. The starved cells must produce nutrition by themselves; otherwise, they would die owing to lack of nutrition, which should decrease of the surface potential. The interfacial pH should have been moderated to the pH in the bulk solution as a result of diffusion because dead cells do not undergo respiration. On the other hand, the surface potential for HeLa cells after exchanging the culture medium for one containing both serum and glucose also increased steadily owing to the enhancement of cellular respiration caused by the introduction of these nutrients. However, the surface potential shift for the starved cells was unexpectedly larger than that for the non-starved ones. The difference between the surface potentials for the starved and non-starved cells was clearly confirmed by at least seven measurements (Fig. 2(b)), which was evaluated from each peak potential at the peak time (at about 15 h). Thus, a semiconductor-based biosensing device can continuously monitor cellular respiration activity as the interfacial pH, which depends on the cell conditions.

**Figure 3:**
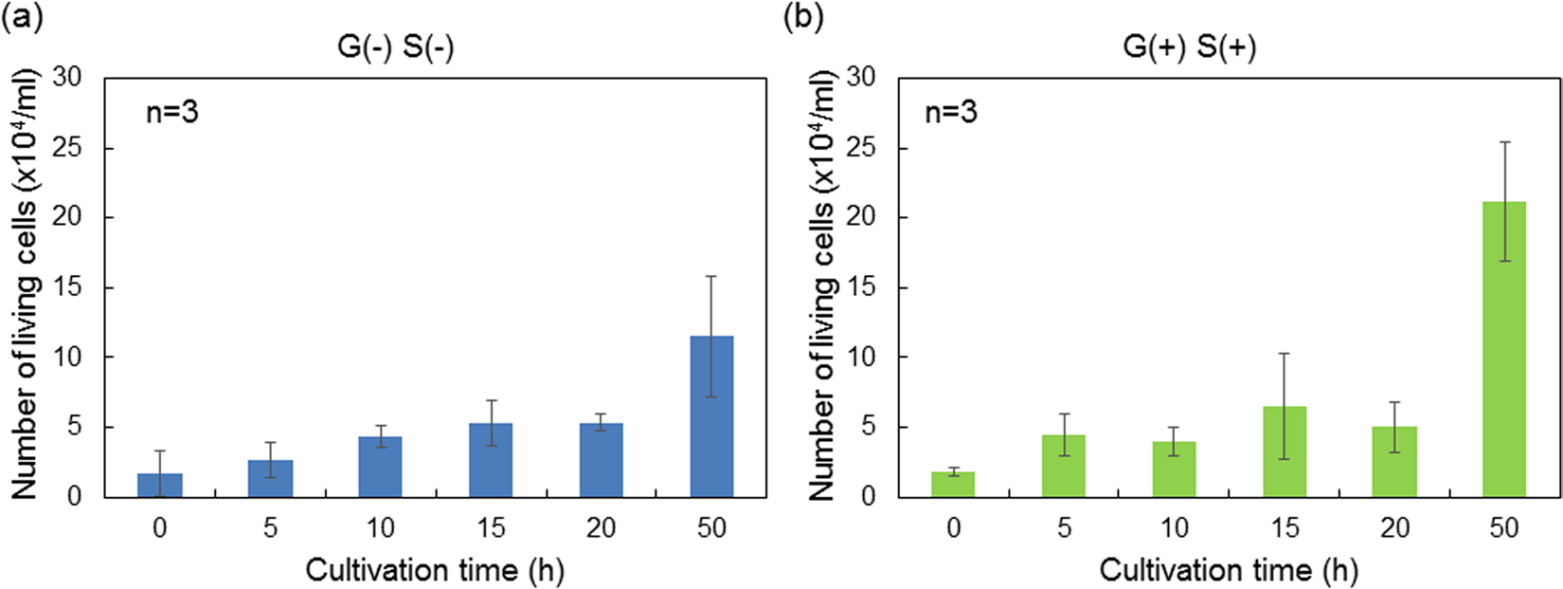
Number of living cells plotted against culture time. (**a**) Number of starved cells in the culture medium without glucose and serum [G(−)S(−)]. (**b**) Number of non-starved cells in the culture medium including glucose and serum [G(+)S(+)]. The culture medium was replaced with a flesh one at 0 h.

In this case, the number of cells adhered on the gate surface was determined for the starved and non-starved cells to investigate the effect of cell proliferation on electrical signals, as shown in Fig. 3. Here, we found that the numbers of all groups showed the same trend until 20 h regardless of the presence or absence of nutrients in the culture medium. However, the change in the surface potential of FET biosensors for starved cells was larger than that for nonstarved cells. This means that the electrical signals provided the basis for the quantitative analysis of autophagic cells as a change in pH at the cell/gate interface. That is, the invisible information of starved cells, which cannot be observed by microscopy, was quantitatively clarified by using the FET biosensors, as shown in Fig. 2, although it was not expected from the number of living cells shown in Fig. 3. Therefore, the respiration activity of starved cells was found to be higher than that of nonstarved cells. The number of starved cells did not increase to a greater extent than that of nonstarved cells at 50 h (Fig. 3) because of the lack of nutrients available for the proliferating cells, whereas the viability of nonstarved cells was hardly reduced even at 50 h; this may explain why the surface potential for the FET with the nonstarved cells did not decrease (Fig. 2).

**Figure 4:**
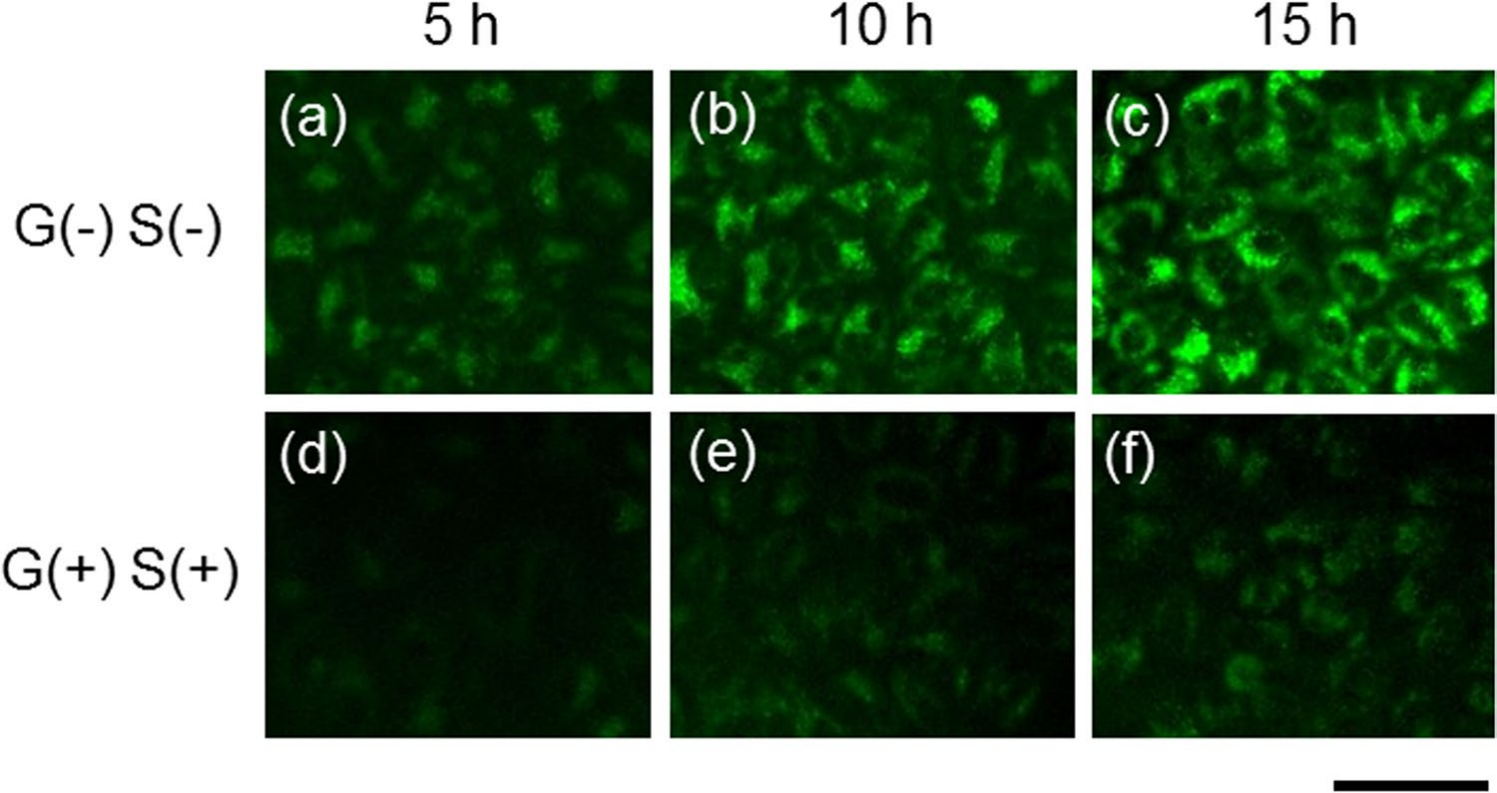
Fluorescence images of autophagic cells. DALGreen was utilized for the imaging of autophagic cells. Starved cells were observed in the culture medium without glucose [G(−)] and serum [S(−)] at (**a**) 5, (**b**) 10, and (**c**) 15 h, whereas the nonstarved cells were observed in the culture medium with glucose [G(+)] and serum [S(+)] at (**d**) 5, (**e**) 10, and (**f**) 15 h. The culture medium was changed with a flesh one at 0 h. Scale bar, 50 μm.

**Figure 5:**
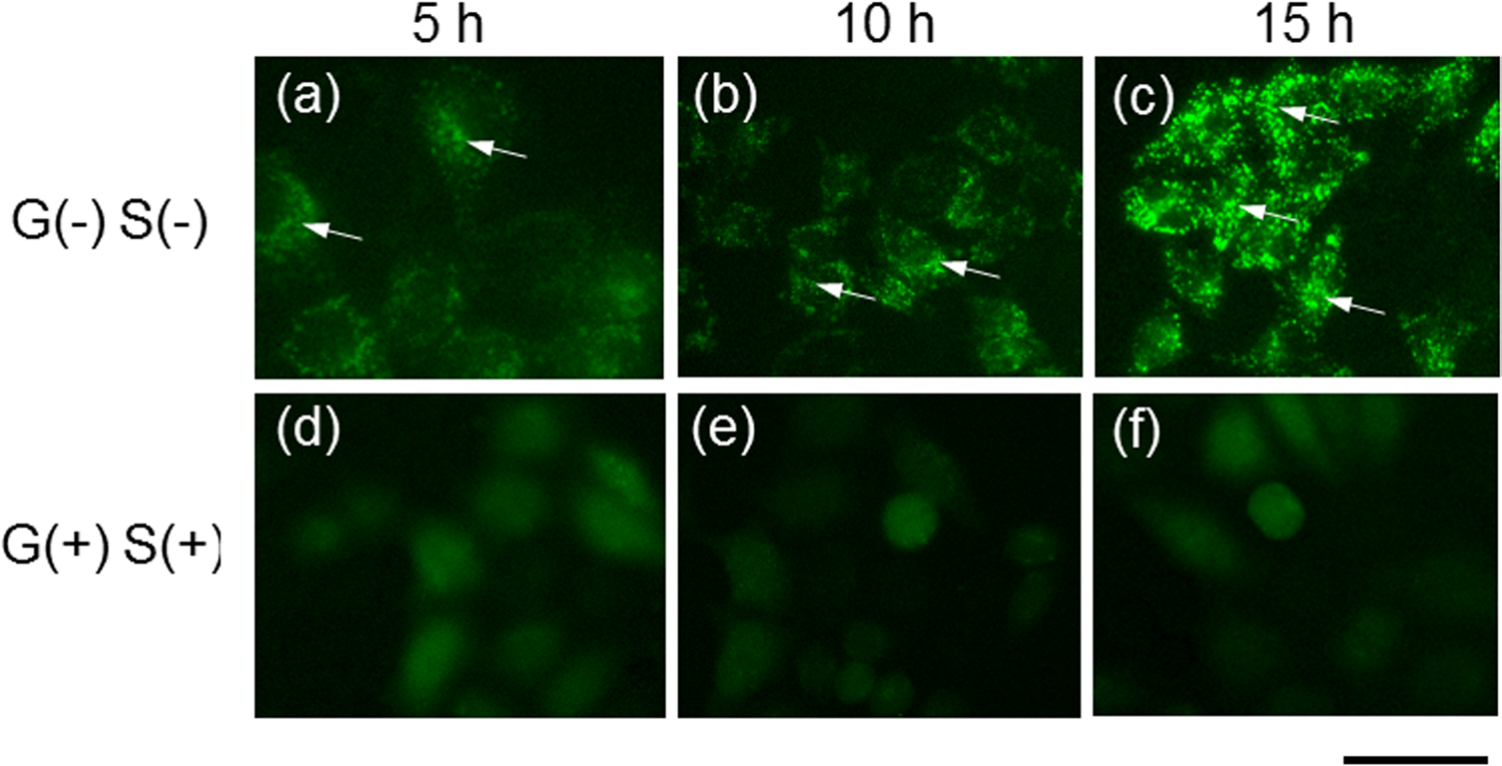
Fluorescence images of autophagic cells in which the GFP-LC3 vector was incorporated. Starved cells were observed in the culture medium without glucose [G(−)] and serum [S(−)] at (**a**) 5, (**b**) 10, and (**c**) 15 h, whereas the nonstarved cells were observed in a culture medium with glucose [G(+)] and serum [S(+)] at (**d**) 5, (**e**) 10, and (**f**) 15 h. The culture medium was replaced with a flesh one at 0 h. In (**a**), (**b**), and (**c**), the formation of green fluorescent puncta was confirmed in starved cells (arrows). Scale bar, 50 μm.

Moreover, autophagy in starved cells was observed using the fluorescent dyes DALGreen and MDC^26^. As shown in Fig. 4, DALGreen was introduced into HeLa cells cultured in the medium without glucose and serum [G(−)S(−)] and in that with glucose and serum [G(+)S(+)]. The fluorescence of the DALGreen dye was clearly observed in the HeLa cells under nutrient starvation [G(−)S(−)]. The images of HeLa cells were obtained at 5, 10, and 20 h after removing their nutrients when the surface potential was increasing owing to autophagy. At the same time, the fluorescence of MDC was also clearly observed in the HeLa cells under nutrient starvation, whereas the existence of dead cells was verified using PI. No PI fluorescence was observed for both starved and nonstarved cells (Fig. S2). Moreover, recent studies on the molecular mechanism of autophagy have led to the development of several marker proteins for autophagosomes, the most widely used of which is LC3, a mammalian homolog of Atg8. These marker proteins allow the identification of autophagic structures by fluorescence microscopy^27,28^. Actually, the formation of green fluorescent puncta was confirmed in the cytosol of starved HeLa cells expressing GFP-LC3, as shown in Fig. 5. Thus, the unexpected electrical signals must have been obtained as a result of autophagy.

## Discussion

The FET biosensor used in this study exhibited a sensitivity of about 58 mV/pH, as shown in Fig. S1(c). The changes in the average peak surface potential for the starved and non-starved cells was about +101 mV and +64 mV about 15 h (peak time) after the change of the medium with the appropriate one, respectively (Fig. 2(a,b)). The pair of experiments on the starved and non-starved cells were repeated seven times. Therefore, the interfacial pH values for the starved cells and non-starved cells decreased from pH 7.4 to approximately pH 5.7 and 6.3, respectively, because the original pH of the culture medium was about 7.4. These changes in the interfacial pH were larger than expected. Actually, the pH of the culture medium used as the bulk solution changed little (from approximately pH 7.4 to 7.0) even when cells were cultured on the culture dish for 48 h. This is why the *in situ* pH around the cell/gate interface was different from the equilibrium pH in the bulk solution. That is, the interfacial pH around the cell/gate interface was monitored continuously at a single location because hydrogen ions were concentrated around the cell/gate interface, resulting in the change in pH at the gate.

In particular, HeLa cells were cultured confluently on the gate surface so that the localized hydrogen ions associated with the cellular respiration activity were trapped in the limited space around the cell/gate interface. When the interfacial pH is detected, it is important to consider the localized area surrounded by the cell membrane and gate surface. The interfacial pH is based on the quasiequilibrium interaction of hydrogen ions with hydroxyl groups at the gate oxide, which is different from the pH in a bulk solution; thus, the change in the interfacial pH should be directly detected in a real-time manner. Thus, the localized pH around the cell/gate interface can be monitored by *in situ* measurement using FET biosensors. In particular, the sensing surface of the gate of a FET biosensor is mostly composed of an oxide membrane with hydroxyl groups in the solution, which is sensitive to a change in the concentration of hydrogen ions. Therefore, the cellular respiration activity caused by autophagy under nutrient starvation can be monitored using the principle of semiconductor-based biosensors. Moreover, we found that cellular respiration was more activated by autophagy under nutrient starvation because the amino acids that decomposed from proteins in autophagic cells would have been rapidly spent in cellular respiration through metabolic pathways, such as the metabolic degradation of amino acids and the tricarboxylic acid (TCA) cycle.

## Conclusions

In this study, the metabolism of starved cells, autophagy, was monitored in real time and noninvasively using a semiconductor-based FET biosensor because the sensing surface of the gate of the FET biosensor was sensitive to changes in the concentration of hydrogen ions, and the interfacial pH between the cell and the gate was directly detected on the basis of the non-equilibrium reaction with hydroxyl groups of hydrogen ions at the oxide surface. This is why amino acids that decomposed from proteins in a cell as a result of autophagy under nutrient starvation would have been rapidly spent in cellular respiration. Thus, a platform based on a FET biosensor is suitable for the real-time, non-invasive and label-free monitoring of autophagy related to various diseases in the field of drug discovery and so forth. The effect of autophagy activators such as rapamycin on the electrical signals of FET biosensors will be investigated in the future.

## Materials and Methods

### Electrical measurement using FET

The detection principle of a FET biosensor is based on the potentiometric detection of changes in charge density at the gate electrode, on which hydrogen ions interact with hydroxyl groups at the oxide surface in solutions according to the pH and specific binding between target and probe molecules occurs for molecular recognition. Basically, ionic or molecular charges at the gate interact electrostatically with electrons in silicon crystals across the thin gate insulator and induce electrical signals owing to the field effect, resulting in changes in the source current and drain current (I_D_) at the channel (Fig. S1). The electrical characteristics of a FET sensor, such as the gate voltage (V_G_)-drain current (I_D_) characteristics and the surface potential at the gate surface, were measured in each buffer solution (pHs 1.68, 4.01, 6.86, 7.41, and 9.18; Wako) using a semiconductor parameter analyzer (B1500A, Agilent) and a real-time potentiometric analyzer (U2723A, Agilent), respectively. As the basic electrical characteristic, the threshold voltage shift ΔV_T_ was defined as the difference in the V_G_-I_D_ characteristics at a constant I_D_ of 1 mA. The time course of the surface potential at the gate surface was monitored using a circuit^29^ with which the potential change at the interface between an aqueous solution and a gate insulator can be read out directly at a constant I_D_. In the present study, the drain voltage V_D_ and I_D_ were set to 2.5 V and 1 mA, respectively.

### Cell culture and nutrient starvation

HeLa cells were cultured on a cell culture dish at 37 °C with 5% CO_2_ in the incubator system. Dulbecco’s Modified Eagle Medium (DMEM, Invitrogen) (including 10% fetal bovine serum (FBS) and 1500 mg/ml glucose) was prepared as the culture medium. The number of seeded cells was about 5 × 10^4^ cells/ml for all the experiments. After the preculture, HeLa cells were seeded on the gate surface of a FET biosensor, on which a glass ring with a diameter of 10 mm was fixed for cell culture using polydimethylsiloxane (PDMS), and the cells were cultured on it under suitable conditions for 1 day in an incubator. The FET biosensor with HeLa cells was set up for electrical measurement in an incubator system including a microscopy system at 37 °C. After confirming that signal traces were almost steady, the culture medium was exchanged under two conditions; one experiment was performed using a prepared culture medium including serum and glucose and the other was performed using the original culture medium without serum and glucose to examine the effect of nutrient starvation on electrical signals generated during cell culture. We set a standard of signal drift less than mV orders per hour as a base line of steady potential. Autophagic cells were confirmed by introducing the fluorescent dye monodansylcadaverine (MDC, Cayman Chemical)^26^ and DALGreen (DALGreen – Autophagy Detection, Dojindo), and by introducing green fluorescent protein - light chain 3 (GFP-LC3, Merck Millipore) into cells^27,28^, while cell death was observed using the fluorescent dye propidium iodide (PI, Wako). GFP-LC3 was introduced into cells in accordance with the protocol of the commercialized product (Merck Millipore). All fluorescence measurements were performed for HeLa cells cultured on conventional cell culture dishes to capture clear images by fluorescence microscopy (Keyence). The number of living cells was counted using a hemocytometer excluding dead cells stained by trypan blue (Dojindo).

## Electronic supplementary material

Supplementary Information
