# Supplementary material for: In situ measurement of autophagy under nutrient starvation based on interfacial pH sensing
Source: Sci Rep. 2018 May 29;8:8282. doi: 10.1038/s41598-018-26719-4 (PMC5973944; doi:10.1038/s41598-018-26719-4)
Supplement: Supplementary file 1 — Supplementary Information [file 41598_2018_26719_MOESM1_ESM.pdf]

In situ measurement of autophagy under nutrient starvation based on interfacial pH sensing

Supplementary Information

Toshiya Sakata*, Akiko Saito & Haruyo Sugimoto

Department of Materials Engineering, School of Engineering, The University of Tokyo, 7-3-1 Hongo, Bunkyo-ku, Tokyo, Japan 113-8656

*Corresponding author. E-mail: sakata@biofet.t.u-tokyo.ac.jp

S1. Detection principle of ion sensitive field-effect transistor (FET)

Figure S1 Concept of FET biosensor. (a) Schematic illustration of FET biosensor. The gate insulator was composed of Ta_2O_5, Si_3N_4 and SiO_2 as shown in (b), each thickness of which was 100 nm, 100 nm and 50 nm. (b) Solution/gate insulator interface. Oxide surface is mostly covered by hydroxyl groups in solutions, which interact with hydrogen ions as equilibrium reaction. (c) pH response of FET biosensor. pH was exchanged from 1.68 to 9.18 in turn respectively, as shown in the left graph. Measurement solution was exchanged to next buffer solution at the point of arrow. Correlation between gate voltage and pH corresponding to pH response is shown in the right graph. The averaged gate voltages were calculated and plotted for the last 1 minutes in each pH response. The gate voltage for pH variation showed about 58 mV/pH near Nernsian response at room temperature.

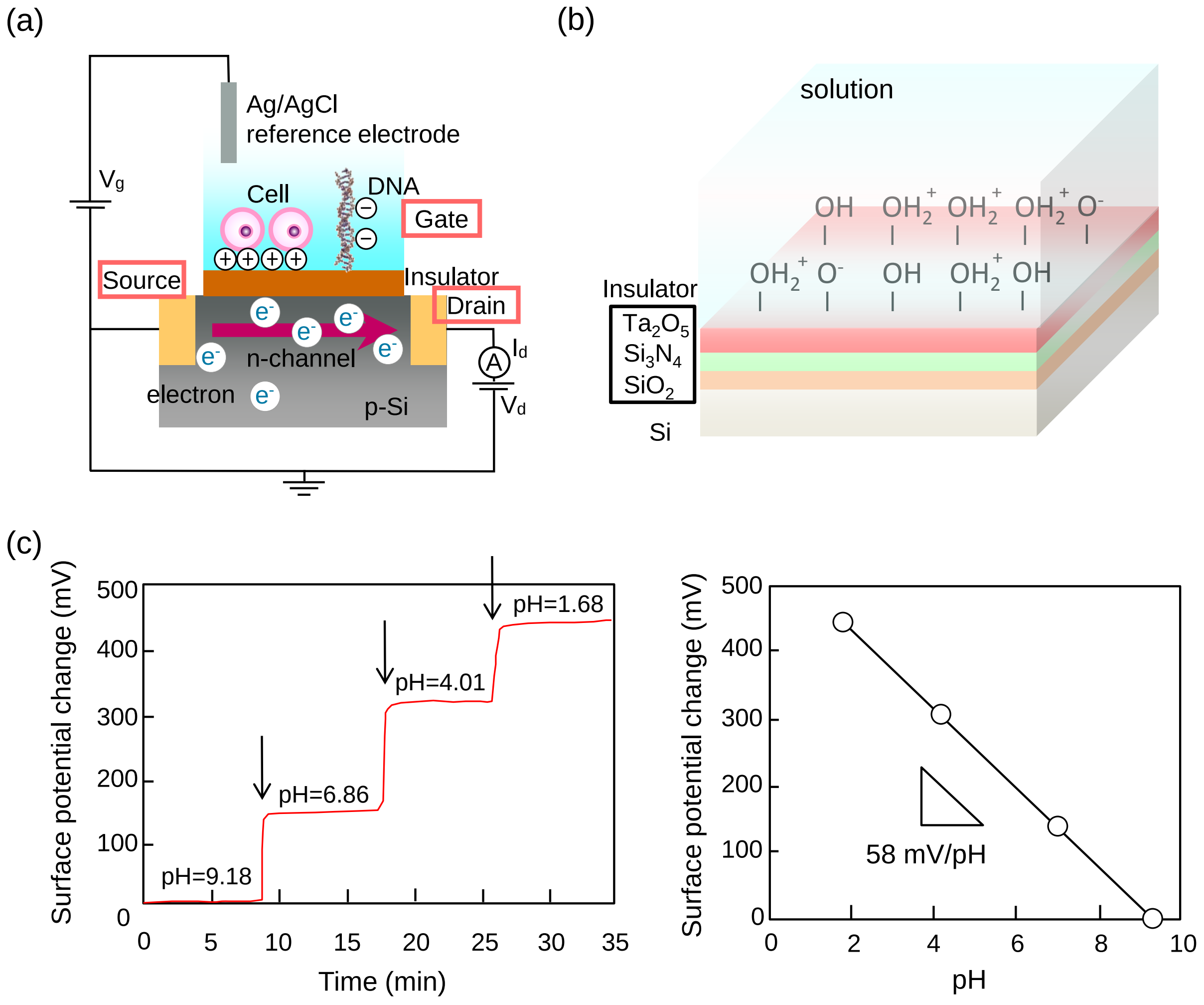

Figure S1

S2. Fluorescence analysis of autophagy using monodansylcadaverine (MDC)

Figure S2 Fluorescence images of autophagic and dead cells. The fluorescent dye monodansylcadaverine (MDC) was utilized for the imaging of autophagic cells ((a) and (b)). On the other hand, propidium iodide (PI) was introduced into the same cells to confirm cell death ((c) and (d)). The starved cells were observed in the culture medium without glucose (G(-)) and serum (S(-)), as shown in (b) and (d), while the non-starved cells were observed in a culture medium with glucose (G(+)) and serum (S(+)), as shown in (a) and (c). Scale bar, 100 μm.

The MDC dye was introduced into HeLa cells cultured in the media without glucose and serum (G(-)S(-)) and with glucose and serum (G(+)S(+)). The fluorescence of the MDC dye was clearly observed in the HeLa cells under nutrient starvation. The images were obtained for HeLa cells 20 h after removing their nutrients when the surface potential was increasing owing to autophagy. At the same time, the existence of dead cells was verified using PI fluorescent dye. Fluorescence based on PI was not almost observed for both starved and non-starved cells.

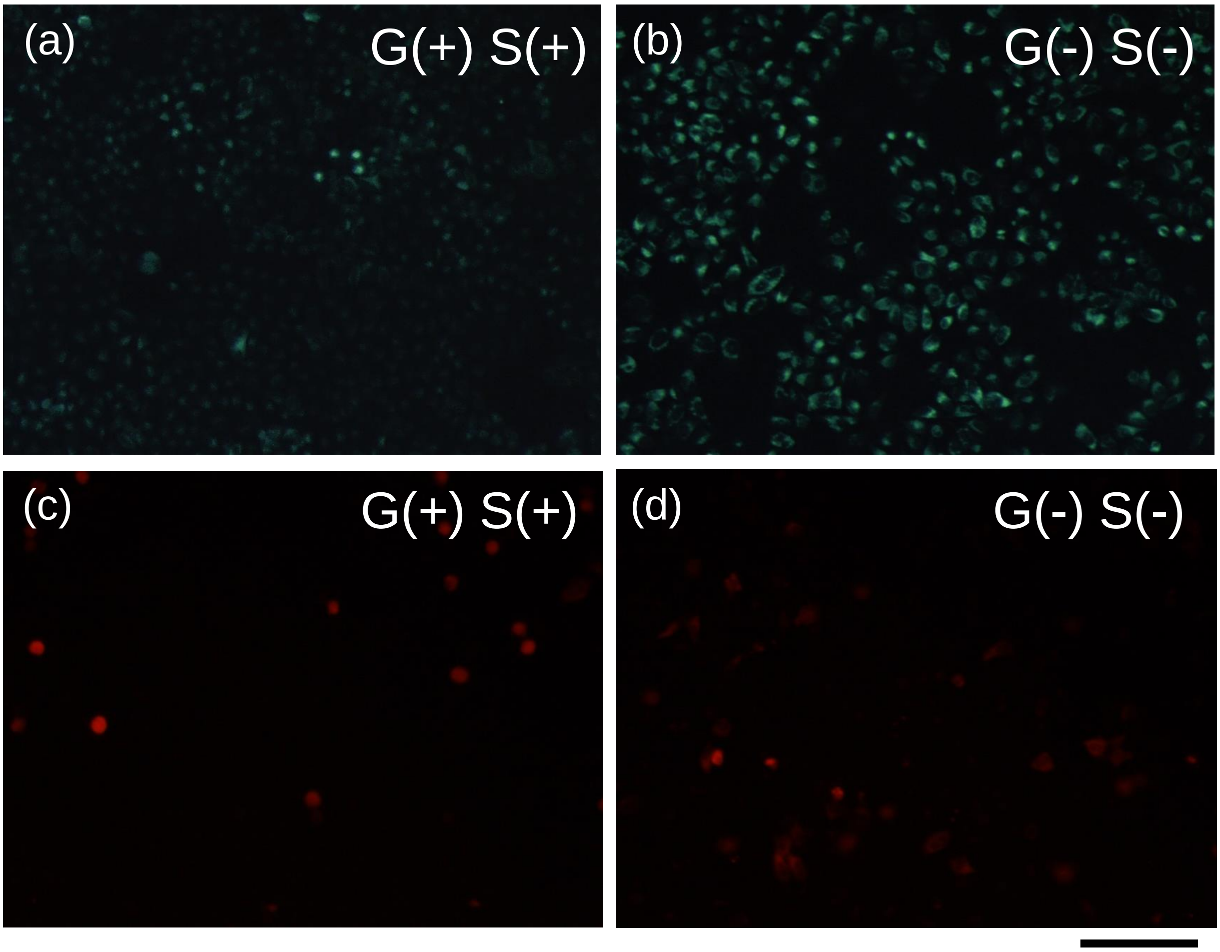

Figure S2
